# A Review of the Concept of Time Reversal and the Direction of Time

**DOI:** 10.3390/e26070563

**Published:** 2024-06-30

**Authors:** Cristian López, Olimpia Lombardi

**Affiliations:** 1 Department of Philosophy, University of Lausanne, 1015 Lausanne, Switzerland; 2 National Scientific and Technical Research Council of Argentina (CONICET), Buenos Aires 1406, Argentina; olimpiafilo@gmail.com

**Keywords:** time reversal, motion reversal, time asymmetry, symmetry, the arrow of time

## Abstract

**Abstract:** In the debate about the direction of time in physics, the concept of time reversal has been central. Tradition has it that time-reversal invariant laws are sufficient to state that the direction of time is non-fundamental or emergent. In this paper, we review some of the debates that have gravitated around the concept of time reversal and its relation to the direction of time. We also clarify some of the central concepts involved, showing that the very concept of time reversal is more complex than frequently thought.

## 1. Introduction

There is a widespread tradition in philosophy and physics that links the problem of the direction of time with the concept of time-reversal invariance. The idea is basically that if the fundamental dynamical equations are time-reversal-invariant, this is a sufficient condition to deem the direction of time as non-fundamental (or emergent, or derivative). This means that if fundamental laws of physics are time-reversal-invariant, then there is nothing in the behavior of fundamental physical systems that singles out a unique directionality of time. This would therefore be just a macroscopic or derivative feature of the natural world ([1,2,3,4,5,6,7] among others). At the fundamental level, reality lacks a temporal direction, at least as we experience it.

The rationale suggests that physical symmetries (in this case, time-reversal symmetry) are somehow informative with respect to the nature of the world (in this case, whether it comes equipped with a direction of time). This inference has been supported by many philosophers and physicists in recent years, so we do not put it into question here (see [8,9,10,11,12,13,14]; for criticisms, see [15,16,17]). We rather focus on (most) laws of physics being time-reversal-invariant as a premise in this argument. But what does it mean exactly to time-reverse a dynamical law (or a physical system)? In this clarificatory paper, we want to flag that time-reversal invariance is not like the rest of the spatial-temporal symmetries. We argue that it is entangled not only with various conceptual problems around what it is meant by time, but also with problems around its formal implementation across physical theories. The main problem that we want to draw attention to is that the concepts of time reversal, time evolution, time translation, and motion reversal are frequently mixed, causing a great deal of confusion. We thus submit that time-reversal invariance is special, both for conceptual and formal reasons.

The target of our paper is first and foremost a common view in philosophy and physics, which we shall call ‘the orthodox view’. According to this view, it is not only the case that time-reversal invariance and the direction of time are somehow connected, but also that the laws of physics are relevant for addressing the problem of the direction of time. We do not want to debunk that view, but rather to clarify many of the concepts involved and the numerous subtleties that it features. The structure of the paper is as follows. In Section 2, some of the complexities of time reversal and its relation to the direction of time are introduced. In Section 3, a first approach to time reversal is undertaken, in both its conceptual and formal aspects. In Section 4, a second, refined approach is developed, in which a distinction between time translation and time evolution, as well as between motion reversal and time reversal, is carried out. In Section 5, some subtleties about how to pick a time reversal operator are explored, focusing on non-relativistic quantum mechanics. Finally, in Section 6 and as final remarks, we take stock on the debate and mention alternative views.

## 2. Time and the Direction of Time

At an intuitive level, there seems to be a substantial difference between space and time. To begin, we can move around space freely (up and down, left and right, etc.), but we cannot do the same with time (we can only “move” forward, so to speak). Our languages also capture such a difference. Natural languages are, in general, tensed languages, in the sense that they have grammatical means to indicate whether an event or action occurs in the future, in the past or simultaneously with respect to a speaker. Philosophers talk about tensed languages as locating events or actions within a dynamic series (a so-called ‘A-series’; see [18,19,20,21]), in which each instant does not only “flow”, but is also located in the past, present, or future. We also capture such a difference between space and future epistemically in assigning some mental states to the past (as memories), and others to the future (as expectations). It would be awkward to say that we can remember something that has not happened yet, as well as to say that we can expect something to happen when it has already happened (see [22]). Note that, at a linguistic and an epistemic level, we do not draw the same grammatical distinctions with space.

The differences between space and time can be also deeper. It could be argued that our knowledge itself presupposes such differences. The German philosopher Immanuel Kant famously argues that space and time are different; even though they are pure forms of sensibility (that is, they are a priori conditions for having intuitions [23] (B37–B73), time is the pure form of the inner sense, and space of the outer sense. They share the property of not being external objects, but different idealities that refer to the subjective conditions of possible intuitions by representing phenomena. Yet, their transcendental origin is different. It means, therefore, that our perception itself must necessarily be spatio-temporal. In a more moderate line, it can also be argued that scientific knowledge presupposes the difference between space and time. Richard Healey [24] says that any physical theory, even a quantum theory of gravity, would be empirically incoherent if it deems time as illusory and identical to space (see [25] for a more general case). The basic argument is that the main means to test empirical theories is through temporal experiments, and that our experience of such experiments is also temporal. So, a physical theory that denies substantial differences between space and time is self-defeating and incoherent—the empirical criteria to adopt it should have to be regarded as illusory, so it lacks credible empirical basis.

Physics itself seems to also recognize some differences between space and time, which go beyond a simple report of our knowledge or experience. To begin with, physics studies the behavior of matter in space and time, i.e., how things change in time in terms of laws that give their temporal evolution, rather than their spatial evolution. And even though physics has increasingly deprived time from its intuitive features, it is still somehow special within physics in the sense that the scientific study of space (or the spatial features of things) commands a different approach than the study of time (or the temporal features of things). But, in an important sense, physics highlights temporal aspects as crucial for a complete account of the natural world—we could not explain basic phenomena without a dynamical explanation of them.

We can ask ourselves what we exactly mean by ‘time’ or by ‘temporal aspects’ in these cases. We said that one of the paramount roles of physics is to describe how things change in time. But are change and time the same thing? Since antiquity, there has been a strong motivation to associate time with change. Aristotle famously says that time is the measurement of change (Aristotle 1992: IV, Delta, 11) (for Aristotle, time is not identical to change, however. Time is a measure of change and depends on change, but it is different with respect to change), but change cannot stand alone, since it must always be related to something else—something changes with respect to something else. It is this observation that led G. W. Leibniz to argue that time is merely an idealization (or abstraction) from change, which is grounded in temporal relations (see [26]; for a contemporary Leibnizian approach, see [27]). Yet, modern physics was born with a different concept of time: Newtonian time is independent from change, as it is a substance whose qualities are independent from the behavior of matter ([28]; for a contemporary philosophical defense, see [29,30]). Therefore, it seems that we have, prima facie, two radically different concepts of time: a relational view (mainly represented by Aristotle and Leibniz,) and a substantivalist view (mainly represented by Newton). Both views acknowledge that time and space are different, but they disagree on the nature of time: relational or substantival.

Our focus is not the difference between space and time (for an extensive discussion on this topic, see [5]), nor the nature of time. Our focus is rather the concept of time reversal and its link to the direction of time. But there is a connection: our concept of time reversal depends on the concept of time we have; our concept of the direction of time depends on the concept of time we have as well. Indeed, one of clearest differences between space and time is that the latter is directed, that there is a sharp difference between past and future, while there is no similar difference between, say, right and left. Yet, when we talk about the direction of time, are we talking metaphorically or literally? That is, are we referring to the direction of time itself, or rather to the direction of change? Physics, as we will show later, seems to assume a metaphorical meaning for the direction of time; what is really meant is the direction of physical evolutions that occur in time (that is a wordy way to say that physics means the direction of change: the direction in which the configuration of matter—particles or fields—varies at different times). Nonetheless, this assumption requires further reasoning. I can change my relative position to my chair by approaching the door from my desk; I can then trace my steps back (tracing back my relative position to my chair). There is clearly a direction of change that was inverted—I first went from my desk to the door, and then back from the door to my desk—but was there an inversion of time? As we will argue later in the paper, there seems to be, in some cases, a difference between the direction of time and the direction of change, or what we can call “(ir)reversible processes”.

In the same vein, when we talk about time reversal, are we talking metaphorically or literally? That is, are we referring to the inversion of the direction of time itself, or rather to the inversion of the direction of change (e.g., of the change of relative spatial positions)? There is not only a conceptual difference, we believe, but also a formal difference—the implementation of time reversal when it means the inversion of the direction of time itself might be quite different from the implementation of time reversal when it means the inversion of the direction of change within a physical theory. In turn, these differences can affect the inference from time-reversal invariance to the direction of time; whether a theory indicates time-reversal invariance or not will strongly depend on the formal implementation of time reversal, which will in turn strongly depend on the concept of time that it underlies. And that is why conceptual and formal clarifications of time reversal are so necessary.

## 3. What Does It Mean to Reverse Time?

In the previous section, we have suggested that conceptual divergences about the concept of time reversal could lead to formal divergences about its implementation within a physical theory. Notwithstanding this, there is a canonical way to understand time reversal (and time-reversal invariance) that is of some help as a first approximation.

Time reversal is, prima facie, a symmetry transformation that is defined in terms of the specific transformations that it induces when acting upon states, parameters, and variables of dynamical laws. Most laws involve a time parameter, t, so that time reversal (which we can represent by T) first and foremost induces the mapping

(1) T:t→−t

This is not, however, enough. To begin with, it is important to distinguish between a *formal* symmetry transformation (i.e., its mathematical implementation within the formal apparatus of a physical theory) and its physical correlate (or its ontic meaning; see [13]). The formal apparatus of most physical theories will have numerous formal symmetries, but only some of them will be physically relevant. In the case of time reversal, Paul Davies claims that such characterization (1) is purely mathematical, without physical meaning. So, for it to gain physical meaning, it needs to be identified with processes or physically relevant magnitudes [31] (p. 23). In plain terms, this means that time reversal as a symmetry transformation must induce further transformations than merely a reparameterization of t, as in (1). There is a natural step towards that direction; (1) also *logically* induces changes in the first time-derivative magnitudes, such as velocity in Newtonian mechanics. This is, nonetheless, more complicated than it looks, since the definition of the time-reversal transformation (and thereby its specific form) will essentially depend on the theory and the dynamics at stake. In a general vein, it can be said that a time-reversal transformation is a symmetry transformation, such that

(2) $$\;T:t\to -t\;T:s\to s^{*}\;T:O\to O^{*}\;T:D\to D^{*}$$

This means that, in addition to (1), it acts upon physical states ($T(s)=s^{*}$), physical magnitudes ($T(O)=O^{*}$), and the derivative operators, ($T(D)=D^{*}$) that appear in a dynamical equation of a physical theory. The problem to elucidate is how time reversal behaves in each specific theory.

When is the dynamical equation then time-reversal symmetric? The law L is time-reversal symmetric if and only if L preserves its validity and structural form after the implementation of T, properly defined. But what does it mean that it “preserves its validity and structural form”? By validity, it is usually meant that L is time-reversal symmetric if and only if every solution of L is mapped to a solution of L under T. That is, if L is time-reversal symmetric, then T keeps the space of solutions of L (as [7]) points out, this definition is still insufficient. Symmetry must also preserve some further content beyond formal or physical equivalence. There are many cases in which it is possible to define a symmetry transformation that satisfies this criterion, but where both solutions are evidently different (e.g., because observational content is not preserved). We will leave this problem aside). It follows from this that it is expected that T also keeps the functional relations among the elements of a dynamical equation; that is, it preserves its structure $L\left(s,\;O,D,t\right)=L\left(s^{*},\;O^{*},D^{*},-t\right)$.

This only defines a general time-reversal transformation. In each physical theory, T adopts different forms depending on how it acts upon each element in the dynamical equations. Just to give an example, in Hamiltonian classical mechanics, T becomes, say, $T^{H}$. It implements time reversal as

(3) $$T^{H}:t\to -t\;{\;\;\;\;\;T}^{H}:p_{i}\to -p_{i}\;\;T^{H}:q_{i}\to q_{i}$$

That is, time reversal reparametrizes the time coordinate, changes the sign of the $p_{i}$ (conjugate momenta), and leaves the $q_{i}$ unchanged (generalized positions). This induces $T^{H}$ to transform the set of all smooth curves $\left(q\left(t\right),\;p\left(t\right)\right)$ through phase space. In turn, it implies that the Hamiltonian is also kept symmetric, ${H(q}_{i},\;p_{i})={H(q}_{i},\;{-p}_{i})$. As we said before, the Hamiltonian equations of motion will be time-reversal symmetric if $T^{H}$ keeps their space of solutions and structural form.

We have presented, on the one hand, a first approach to the concept of time reversal and, on the other, a formal approach to the implementation of time reversal. Let us now be a bit more precise on this.

## 4. Time Reversal and Time-Reversal Symmetry

Time reversal is interesting per se, but one of the reasons to study it is because of its alleged connection with the direction of time. But what exactly do we mean by the asymmetry or the direction of time in the philosophy of physics? To begin with, when talking about the asymmetry of time or the direction of time, we do not primarily and without further arguments refer to the asymmetry or direction of things in time (see [2]). What we wonder is whether time (however we understand its nature) is literally asymmetric or exhibits a directionality. Second, the asymmetry of time refers to a difference between past and future, while the direction of time refers to the specification of a direction (either past-to-future or future-to-past) on the basis of such an asymmetry. If an asymmetry can be identified, then we can conventionally define a direction, since past and future must be different (see [32]). In what follows, we will refer to the asymmetry of time and the direction of time indistinctly (more technically, it can be said that a direction of time means the existence of a time-like vector field, $t^{a}$ (a temporal orientation), while the asymmetry of time refers to the determination of a choice between $t^{a}$ and ${-t}^{a}).$

Metaphysicians wonder whether time is fundamentally asymmetric. That is, whether reality at bottom is temporally asymmetric or whether it is an emergent (or supervenient) feature of the non-fundamental reality. For instance, philosophers that defend the so-called A- and B-theories of time (for A-theories, see [18,19,20,21]; for B-theories, [33,34,35,36]) will argue that time is intrinsically asymmetric, as instants (or events) are ordered either by absolute monadic predicates that place instants or events in a flowing series from the past to the future (A-theories) or by dyadic relations of “…before than…”/“…later than…” (B-theories). Both theories already presuppose a directionality of time, and only diverge over whether time is dynamic or static. One of the reasons to promote the asymmetry of time to a fundamental feature of reality is the explanatory gain we obtain when accounting for our experience. Theories that deny that time is fundamentally directed (such as the so-called C-theories of time, see [37,38]) bear the burden of explaining why reality looks temporally asymmetric when it is really not. Difficulties in bridging this explanatory gap have led some philosophers to endorse either A- or B-theories of time.

In addition to the value of metaphysical debates around time, physicists and philosophers of physics take another path (by physics here we mainly refer to *theoretical* physics and not to experimental physics. For simplicity, we will use the word physics from now on). The assumption is that our experience of time can ultimately be explained by some material asymmetry in the world. That is, we have the experience of time we have *because* some material asymmetry in the world obtains and can be correlated to our temporal experience [22]. Therefore, if we want to explain our experience of time in the vein of physicalism (that is, in terms of what physical theories say about the behavior of matter), then we have to look at physics in order to see whether it somehow needs to include a temporal asymmetry in its explanations of physical phenomena. As we said in Section 1, it seems blatant, prima facie, that physicalist explanations must include this. But the problem is a bit more complicated, since it might be the case that, although physics aims to explain and describe the changes of things, it can assume that an asymmetry of time is not needed as the basis of its explanations. In one way or another, it is reasonable to believe, in the vein of a naturalistic–physicalist trend, that whether time is asymmetric or not is a question that rests on physics’ shoulders, awaiting a response.

But what should we look at when turning to physics for answers about the asymmetry or the direction of time? There are basically two paths. The first one, “the *orthodox view*”, states that we should look at the dynamical laws of physical theories and see whether they are time-reversal-symmetric. The second one, “the *heretic view*”, states that we should, rather, look at models of the universe (as given by the general theory of relativity, for instance) and see whether they exhibit a time asymmetry in their space-time’s structure (see [6,39,40,41]). This might resemble the distinction between temporal asymmetry at the level of the laws (“laws-first view”) and at the level of solutions (“solution-first view”) (see [32] for details). But the distinction we want to point out here is more specific, since it involves the dynamical behavior of local systems (in which the concept of time-reversal invariance is more important) versus the structure of space-time at a global level as delivered by cosmology and general relativity. In what follows we will focus on the first path, as it is the one that directly concerns the concept of time-reversal invariance. But let us explain a bit further some general differences between both views.

To begin with, the orthodox view considers that it is the set of solutions of equations of motion that is relevant for the asymmetry of time. To put it differently, it does not concern whether an individual solution is time-reversal-symmetric, but whether the space of all solutions of a physical law is time-reversal-symmetric. In addition, this view can be labeled as ‘local’ since it entertains the nomological descriptions of subsystems of a larger system (potentially, the universe). Physical theories such as classical mechanics (in all its formulations), non-relativistic quantum mechanics, quantum field theory, etc., are not theories of the universe per se, but theories of subsystems of the universe (see [41]). In opposition, the heretic view mainly concerns specific solutions of Einstein’s field equations plus their boundary conditions, and asks whether such an individual solution comes equipped with a direction of time. The concept of time-reversal symmetry barely plays a role here, since the asymmetry of time can be grounded not in the laws, but in other non-dynamical elements of a model of the universe (e.g., an asymmetry in boundary conditions or in the geometrical structure of space-time). In addition, this view is genuinely global, as it concerns the properties of the universe as a whole and not of subsystems.

Thus, the orthodox view primarily concerns dynamical laws and their property of being time-reversal symmetric (or not). But which laws? For instance, whatever goes by Newtonian classical mechanics involves a wild number of general laws, particular laws, and models. So it is reasonable to ask which kinds of laws are the relevant ones when asking about time-reversal invariance. Tradition has it that the most general and fundamental laws of a physical theory are the relevant ones for assessing time-reversal invariance and the direction of time (see [42]); that is, the laws that describe free-fall physical systems, abstracting away any non-conservative interaction with the environment. Those ideal laws, it is argued, describe the universe at bottom, as they were the architectonic scaffolding of physical theories. Particular laws are just instantiations of such general laws that introduce additional elements that depend on the particular physical situation and cannot therefore be generalized.

An additional argument to defend this stance is to say that there also seems to be a correlation between such general, ideal laws and the structure of space-time. John Earman [8] and Jill North [43] have argued that physical laws are not written in the air, but on the basis of an underlying space–temporal structure. It follows from this that there must ideally be a matching between the symmetries of the dynamics and the symmetries of space-time ([43,44,45] for an interpretation). This additional argument truly makes sense because it is a clever way to connect the dynamics, on the one side, and the geometry, on the other, and it makes clearer why questions about the structure of time (whether it is directed) can be addressed to the dynamical equations. Dettlef Dürr and Stephan Teufel illustrate this relation by saying that the space-time symmetries are as “the rules of the theater” on which the dynamics will unfold [46] (p. 44) and must respect. (This view can be challenged. Some philosophers have argued that it is unwarranted to privilege general, ideal laws (or models) to draw philosophical conclusions. For instance, Nancy Cartwright [47] argues that general, ideal laws (what she calls ‘covering laws’) do not describe real physical systems, but are actually schema to be filled in with physical content. Keit Hutchison [48] claims that whether a physical symmetry holds or not depends on the force that we are considering. But in such general laws all forces have been abstracted away, so the idea of a physically meaningful symmetry does not even apply. Cristian Lopez [49,50] shows that this is an ontological assumption that should be duly justified because it imposes an ontological imbalance that cannot be justified within a theory.)

A more intuitive argument runs as follows. Many space-time symmetries seem to have an empirical correlate in experiments. Think of space-translation invariance. It basically means that, under some assumptions and abstractions, it does not matter where an experiment is carried out because the symmetry guarantees us that the specific location in space should not affect the validity and the structure of the laws; therefore, it should not affect, ceteris paribus, the experimental results either. The same goes for time-translation invariance and other space-time symmetries. Of course, for this statement to be true is necessary to abstract away the influences of local conditions (i.e., whether the experiment is being carried out under the influence of, say, an inhomogeneous electromagnetic field), but the general claim still holds—variation can only depend on local circumstances because the *general* law holds in general.

We can wonder whether all this reasoning applies smoothly to time-reversal invariance, as well. Our view is that there are good reasons to think it does not, and this is partially why time-reversal invariance is special. First, time-reversal invariance has some empirical constraints, as we mentioned above. While other space-time symmetries can be grasped, and even tested, empirically, we cannot go backward in time. It is even tough to understand what it means to move ourselves backward in time. So, some reasoning that straightforwardly applies to other symmetries could be harder, or impossible, to apply in the case of time reversal. Second, time reversal is not even formally similar to other space-time symmetries. To begin with, it does not belong to the Galilei or Poincaré Group (as many of the others do). But its specific content, both conceptual and formal, is difficult to define as it might not only vary from theory to theory, but also be motivated by different intuitions about what it means. In some cases, there seem to be alternative ways to define time reversal given this ambiguity in its conceptual content. The take-home message is then that time reversal (and time-reversal invariance) is not only special compared to other space-time symmetries, but also imposes further challenges with respect to them.

## 5. How Is Time Reversal Formally Implemented? The Case of Non-Relativistic Quantum Mechanics

All these problems lead to the following: how to pick the right time-reversal transformation. That is, the time-reversal transformation that does what we exactly want it to do—to reverse time. But, as we have anticipated many times, the conceptual divergences upon what reversing time is may induce divergences upon their formal implementations.

A time-reversal transformation should, to begin with, implement an *involution*. That is, a transformation such that, when applied twice, is equal to the identity $T^{2}=I$. This sounds reasonable, but it is not enough to obtain the full content of what it is meant by reversing time. Eugene Wigner [51] says that time reversal is an involution that comprehends a series of operations that must be performed in sequence to obtain the identity

(4) $$T\left[U_{∆t_{2}}T\left(U_{∆t_{1}}s_{0}\right)\right]=s_{0}$$

That is, it is a composite transformation, such that

time displacement by *t* × time reversal × time displacement by *t* × time reversal = *I*

Wigner’s definition of time reversal not only goes beyond a simple involution, but also does not conceive of time reversal as a mere reflection along the temporal axis, as we suggested before when saying that time reversal also reverses first time-derivate magnitudes. Therefore, time reversal is defined as a transformation that must satisfy (4).

Three comments are in order. First, it is now clearer why time reversal is required to invert the sign of other dynamically relevant magnitudes. If the time-reversal transformation is defined by (4), then it must involve transformations that can generate the second time displacement, that is, ones which track the system backward after the first time inversion [49]. This point is important, and we will come back to it later. But, under Wigner’s understanding of time reversal, it could never be a mere reparameterization of t because it would not adjust itself to the definition.

Second, there might be some ambiguity in Wigner’s definition as to what it is meant by ‘time displacement’. We said before that time reversal was relevant for the direction of time and applied to dynamical laws. Now, Wigner’s definition introduces the idea of time displacement in the very definition of time reversal. But time displacement (or time translation) and dynamical evolution (or dynamical law) are different. We can then ask ourselves: do we want time reversal to time *displace* a system backward, or do we want the dynamical law to *evolve* the physical system backward in time? Time displacement (or time translation) is a geometrical operation that moves a system to a different location in time. Time evolution, in turn, is generated by the dynamical law (or the evolution operator in the theory). In some cases, they might coincide (for instance, when physical systems are completely isolated), but in general they do not only represent different concepts, but are produced differently. To illustrate this point, we can take a look at the Galilei Group on non-relativistic quantum mechanics (for further details, see [52]); when quantum systems are isolated, the Galilei Group and its commutation relations imply that time displacement commutes with space rotations, which can be expressed as the commutation between the three angular momentum components and the Hamiltonian. In this case, when the system is isolated, the Hamiltonian (which generates the time evolution) is the time-displacement generator. But when external fields are coupled to the system, this is no longer the case; the Hamiltonian no longer commutes with the three angular momentum operators, but time displacement does (see [53,54]). This means that in these cases, the Hamiltonian retains its role as the generator of time evolution, but is no longer the generator of time displacement.

This comment makes the relevance of our question above clearer. When time is reversed according to Wigner’s definition, we can either be commanding the time *displacement* of the system backward or the time evolution of the system backward. That is, we could be either commanding a geometrical transformation or a dynamical evolution. This ambiguity, we submit, is due to a confusion between time-translation and time-evolution, probably inherited from classical physics, where both concepts are equivalent. But let us give another example to make this case clearer. Think of a space-reversal transformation, defined *à la* Wigner. So, we define a space-reversal operator, X, such that

Space displacement by ∆x × space reversal × Space displacement by ∆x×spacereversal=I

where $∆x=x_{2}-x_{1}$. Now we may wonder whether a physical law L is space-reversal-invariant according to X. Then, we imagine the following sequence: from $x_{1}$ to $x_{2}$ the system evolves (in time) according to the law L; then, at $x_{2}$, the system is space-reversed and translated in space back to $x_{1}$, and space reversal is applied again. The question is then as follows: does the spatial translation from $x_{2}\to x_{1}$ represent a possible evolution according to L? It is clear in this case that there are two different concepts: one is the time evolution that generates a spatial displacement from $x_{1}\to x_{2}$, and the other is a different one that space-displaces the system back from $x_{2}\to x_{1}$. And then we ask whether this space translation is a solution of L or not. Suppose that L is not invariant under space reversal because there is a spontaneous movement of things towards the +x direction of space. It is clear then that, under space reversal, the system can be geometrically space-displaced from $x_{2}\to x_{1}$ (spatially translated “against the current”), but this is not a solution to L because it picks a privileged direction of space, namely, +x. In this case, it would be very clear that evolution and translation are two different concepts that must be kept distinguished so as to recognize cases like this.

To recapitulate our original question, when Wigner says that time reversal involves two time displacements, does he mean by time displacement time evolution according to the dynamical law, or time displacement according to a geometrical transformation? It seems that, conforming with the orthodox view, time evolution is the relevant concept to assess time-reversal invariance: we want to know whether the second time evolution is possible according to the dynamical law, and not if it is geometrically possible (somehow) to time-translate the system back to the original state. This brings us to the third and final comment: if this is so, then it might be problematic to define time reversal in terms of the time evolution, because this is precisely what we want to know. In other words, if the problem behind the scenes is the problem of time asymmetry, and if the problem is defined in terms of whether dynamical laws are time-reversal-invariant or not, then it makes sense to define time reversal in such a way that its definition is independent of time evolution. Otherwise, it is always possible to claim that a time-reversal transformation that does not generate the second time translation (or, better, time evolution) is ill-defined because it must do it to be definitionally coherent. So, it looks like we are facing a sort of dilemma. On the one hand, we want to know whether a time evolution is a possible solution to a given dynamical law when time reversal is applied, and then we require independent means to define time reversal. On the other, the standard definition already presupposes that the second time translation (or, better, time evolution) must be possible, otherwise the transformation is ill-defined. But this trivializes the original question (see [39,40] for an argument in this line).

The confusion between time displacement and time evolution, and the ambiguity in the Wignerian definition of time reversal, are serious enough to take more care conceptually and formally when dealing with time reversal. But there is another, probably more complicated problem that we mentioned in passing before. Ultimately, we want a formal implementation of a concept of what it is to reverse time. But it is still not clear what is meant by ‘time’. This has already been flagged in the specialized literature, where the scope of *time* reversal is severely nuanced. For instance, William Gibson and Brian Pollard [55] say that

“In this approach we see that no metaphysical notion of reversal of the direction of the flow of time is involved. We are led to consider time reversed processes but not reversal time itself. Although motion reversal and motion reversal invariance would be better names, we shall adhere to the accepted, if imprecise, usage.”

[55] (pp. 177)

In the same vein, Leslie Ballentine [54] warns us that

“The term “time reversal” is misleading, and the operation that is the subject of this section would be more accurately described as motion reversal. We shall continue to use the traditional but less accurate expression “time reversal”, because it is so firmly entrenched.”

[54] (pp. 377)

This adds another layer of complexity to the concept and the formalism of time reversal, as it seems that two different concepts (and symmetry transformations) fall under the same label ‘time reversal’. The distinction between motion reversal and time reversal has already been flagged in the philosophical literature (see [56,57,58,59,60]), and there are arguments in favor and against an identification. On the one hand, the physics literature claims that what physics actually implements (and cares about) is properly called motion reversal, which could serve as a proxy to understand time reversal. On the other hand, the philosophical literature warns us that motion reversal and time reversal cannot be identified without further arguments (see [49,57,59]), but that metaphysical and methodological assumptions are also in play. For instance, Cristian López [49,59] argues that motion reversal and time reversal are, prima facie, not only different concepts, but also lead to different formal implementations that should not be conflated. The identification between the two presupposes a relational metaphysics of time, in which time reduces to change (e.g., change of relative positions of physical systems). If relationalism about time is assumed, then it is reasonable to associate time reversal with motion reversal, because there is nothing else that time reversal could mean aside from the meaning already given by motion reversal.

This distinction arises saliently in non-relativistic quantum mechanics. In that case, the time-reversal transformation is defined by an anti-unitary operator, $T^{Q}$, that

(5) $${\;T}^{Q}:t\to -t\;{\;\;\;\;T}^{Q}:P\to -P\;\;T^{Q}:X\to X\;{\;\;T}^{Q}:\psi \to \psi^{*}\;T^{Q}:i\to -i$$

That is, it reparametrizes the time coordinate, flips the sign of the momentum operator, leaves the position operator unchanged, but more remarkably, takes the complex conjugate over the quantum state (which explains why it is anti-unitary). But why is the time-reversal operator defined in such a way? One answer is that it is the only time-reversal operator that can accommodate Wigner’s definition of time reversal, as introduced above. But, as has been noted, this implementation of time reversal aims to represent *motion* reversal (see [54,55,60]; see [57] for philosophical literature). So, it assumes that either time reversal per se cannot be represented in the theory according to Wigner’s definition or that time reversal *is* motion reversal.

What would happen if time reversal were implemented differently in non-relativistic quantum mechanics? Suppose that an alternative time-reversal operator is defined, say $T^{Q2}$. And that it is defined as in classical physics, namely, as a reparameterization of time that induces changes only on the first-time-derivate magnitudes. This *unitary* operator would not take the complex conjugate over the quantum state, since the state has nothing to do with time per se (see [49,56,57]). Interestingly, such a defined time-reversal operator would fail to accommodate Wigner’s definition of time reversal, since the backward-in-time second time translation (or, better, time evolution) is not a solution of the Schrödinger equation. The reason is that a unitary time-reversal operator would render the time-reversed Hamiltonian of the system unbounded from below, which is physically impossible. So, there seem to be two ways to go: either $T^{Q2}$ is the right formal implementation of time reversal and we reject Wigner’s definition of time reversal as really defining time reversal, or we preserve Wigner’s definition and redefine the time-reversal operator in such a way that meets it (say, as $T^{Q}$).

Both ways have interesting consequences from a conceptual viewpoint. The first one suggests that Wigner’s definition of time reversal is not really defining time reversal, but something different. It could be argued, for instance, that Wigner’s definition gives criteria to implement motion reversal. In some sense, this seems to be what is going on—physicists, and certainly Wigner among them, have good reasons to demand a transformation that keeps the reversion of motion that satisfies the Wignerian definition. In systems isolated from external interactions, an evolution would be physically equivalent if it happened in one direction *and* the other, and that is what Wigner seems to have in mind. Heuristically, it is also reasonable to assume that such a symmetry exists in physical theories (see [61]); asymmetries can then be explained in terms of interactions that break the symmetry, so when an asymmetry appears, physicists have the motivation to look for the interactions that breaks a hypothetical underlying symmetry. That is why some physicists have seen in the Wignerian definition a sort of a priori condition that dynamical laws should have (see, for instance, [46] (pp. 43–44) and [62,63]). But it should be made clear that we are technically talking about *motion*-reversal and not *time-*reversal, the implementation of which is now an enigma. This would of course have philosophical consequences for the problem of the direction of time—the inference as we presented it before was established in terms of time reversal—but it happens that physics actually speaks of motion reversal. It is thus worth asking whether motion reversal is informative with respect to the direction of time.

The second way is to adjust the time-reversal transformation in each case to Wigner’s general definition and to acknowledge that it aims to capture the concept of time reversal. As previously mentioned, some philosophers have argued that this would mean that time reversal and motion reversal are identical, either for pragmatic or conceptual reasons. A pragmatic reason is that physics is not concerned with unempirical claims about time or time reversal. The question of what is time reversal per se would lie in the metaphysical realm, since there is no means to test it as independent from change. A conceptual reason is that if a relational view of time is endorsed (as mentioned in Section 1), then nothing is time-independent from change (i.e., motion), and so time reversal can mean nothing but motion reversal. In other words, since time is reduced to motion, any temporal locution actually refers to facts concerning change. Time reversal must then be interpreted as actually referring to change, which in physics is in general motion. The virtue of this approach is that it is now plain and clear what time reversal is, and it is in accordance with physicists’ doings. The drawback is that it crucially hinges upon a specific philosophical theory of time (relationalism in any of its versions), which could be challenged on both philosophical and scientific grounds.

## 6. Final Remarks: Time Reversal, the Direction of Time and Alternative Views

As was argued in the previous sections, time reversal is special within space-time symmetries not only for theoretical reasons, but also for conceptual ones. We first drew attention to the relation between time and time reversal, and how our understanding of the former affects our understanding of the latter. This was illustrated by the distinction between motion and time reversal, and the alternative representations that this allows (e.g., in non-relativistic quantum mechanics). Then, we also stressed some ambiguities in the Wignerian definition of time reversal as the difference between time translation and time evolution, and whether time reversal can be defined independently from time translation or time evolution.

Let us now go back to our original concern: time reversal and the problem of the direction of time. As we said, tradition has it that the fact that dynamical equations of motion (of a special kind) are time-reversal-invariant is relevant for the problem of the direction of time. There is a well-thought-out inference that links time reversal, on the one hand, with the structure of time on the other. But the problem has become more complex than originally thought, as there is no unique and obvious way to establish when an equation of motion is time-reversal-invariant or not in the relevant sense. This fact, as we reviewed, can be tackled from different perspectives that do not share the unalike assumptions that can lead to different definitions and implementations of time reversal. As a matter of fact, whether an equation of motion is time-reversal-invariant or not depends on the definition of time reversal, and there is no guarantee that different definitions will preserve the invariance of the equation. This happens in, for instance, non-relativistic quantum mechanics and the Schrödinger equation: it is invariant under $T^{Q}$, but non-invariant under $T^{Q2}$. If we then take the inference to heart, then there are reasons to think that there is no direction of time (at least at the level of the fundamental dynamics) if time reversal is implemented as in $T^{Q}$. But the inference no longer goes through with $T^{Q2}$.

This does not mean that the orthodox view, which focuses on the dynamical laws, must be rejected. It means that it must take many more aspects into consideration and argue for them accordingly. Or, this puzzling situation could be a motivation to abandon the orthodox view, at least as it stands. We have mentioned the heretic view as an alternative, but let us mention a slightly different view, which is halfway and branches off from it. The primary intuition about the directionality of time actually comes from *irreversible* processes, such as ice melting at room temperature, human aging, or the expansion of gases in a closed box. It is true that reversibility has been identified with time-reversal invariance, but this is a conceptual confusion, or so it can be argued. Reversibility is a property of a singular solution of a dynamical equation, while time-reversal invariance is a property of the dynamical law (or of its space of solutions). The irreversibility of a solution (or model) can be due to an asymmetry in the boundary conditions and not necessarily because the dynamical law itself is non-time-reversal invariant (for this distinction, see [3,40]). For instance, it has been argued that very special initial conditions can generate an asymmetry that recovers a directionality of time (this is what Huw Price calls “the one asymmetry view”, [64]) without the need for non-time-reversal invariant laws. In the same vein, the postulation of a very low entropy microstate in the past can recover all our epistemic and physical differences between past and future (see [7,56,65,66]). The intuition behind adopting this view is that the problem of the direction of time is in reality a problem of what explains our time-asymmetric experience, and not about the whole set of possible solutions of equations of motion. Entropy-increasing behaviors plus an asymmetric initial condition would be enough to get the explanations right.

This alternative view nonetheless faces serious drawbacks. To begin, it seems to be limited to local regions of the universe, since there is no way to establish from within the universe if two irreversible processes are coordinated in the same direction. When we move to the global scale, it makes sense to ask whether all irreversible processes can be coordinated in the same direction, and then say that the universe as a whole is also irreversible. But this is precisely what cannot be carried out through local laws, or better, through theories of subsystems of a larger system. In addition, it is not clear whether there is a uniform magnitude that exhibits some asymmetric behavior and relates all the phenomena of the universe; many phenomena are irreversible, but it is not clear if they are so in the same sense. For a long time, it was thought that entropy was such a magnitude (see [67,68]), but this view has weakened lately. One of the reasons is that there are plenty of time-asymmetric behaviors, even more fundamental, that are not related whatsoever to entropy (e.g., the decay of neutral kaons or beta particles in weak interactions, see [69,70]).

Therefore, the focus on irreversible processes after the abandonment of time-reversal invariance and dynamical laws solves some problems but incurs others. One of them is the incapability of referring to the global scale and settling a direction of time for the universe. For this case, it seems that the only solution is to abandon the orthodox approach and its alternatives and explore a different path, such as the heretic view. But, in this case, the concept of time reversal becomes much less important than tradition has assumed.

## Data Availability

No new data were created.
